# Angiogenesis in Head and Neck Cancer: A Review of the Literature

**DOI:** 10.1155/2012/358472

**Published:** 2011-11-10

**Authors:** Codecà Carla, Ferrari Daris, Bertuzzi Cecilia, Broggio Francesca, Crepaldi Francesca, Foa Paolo

**Affiliations:** Division of Medical Oncology, A.O. San Paolo Via di Rudinì 8, 20122 Milan, Italy

## Abstract

Angiogenesis is a necessary process for tumor growth, progression and diffusion. In the last years many efforts have been made to understand the mechanisms necessary to the formation of new vessels in tumor tissue and how to integrate these findings in the treatment of different type of cancer. Thanks to these studies there are today many anti-angiogenic drugs with established activity in cancer and approved in clinical practice. Head and neck cancer is a common tumor worldwide that often has advanced stage at diagnosis and poor prognosis. Angiogenesis has a well recognized role in head and neck cancer progression and resistance to drugs and radiotherapy and many clinical trials has been conducted with antiangiogenic agents in this disease, even if they often showed limited efficacy. In this review we summarize the main trials published about angiogenesis in head and neck cancer with particular attention to factors involved in this process and the available data on the efficacy of treatment with anti-angiogenic agents in this disease.

## 1. Introduction

Squamous cell carcinoma of the head and neck (HNSCC) is the sixth most common cancer with 500.000 diagnosis per year worldwide [1]. Patients with locally advanced disease have a chance of cure with multimodality treatments that involves surgery, radiotherapy, chemotherapy, and, in the last years, molecular targeted therapies [2]. Despite the advances in the treatment of locally advanced disease, more than 50% of patients will relapse. Furthermore, combining surgery, radiotherapy, and chemotherapy often leads to severe and permanent function deficits with a negative impact on patients' quality of life. On the other hand, patients with relapsed or metastatic disease have a worse prognosis with an overall survival of approximately 7–10 months [3]. New therapeutic protocols and agents should be developed to improve survival while limiting treatment-related toxicities.

Angiogenesis, the process that leads to the formation of new vessel, is a hallmark of tumor progression, and its role has been studied in many cancer types including HNSCC. Antiangiogenic agents are to date available and useful for the treatment of many tumors. In HNSCC; however, few clinical trials have yielded promising results when focusing on these new agents.

This paper is aimed at evaluating the angiogenic factors involved in HNSCC growth and progression and their therapeutic implications. 

## 2. Angiogenesis in Head and Neck Cancer

Vascular endothelial growth factor A (VEGF-A) is the best known agent that induce angiogenesis. It is a vascular permeability factor that belongs to the platelet-derived growth factor (PDGF) superfamily, which also includes VEGF-B, VEGF-C, VEGF-D, VEGF-E, and placental growth factor (PlGF) [4]. Hypoxia induces VEGF expression through the mediation of hypoxia-inducible factor (HIF-1*α*) [5]. There are many other factors involved in angiogenesis, such as epidermal growth factor (EGF), PDGF, prostaglandins, COX-2, and IL-6 [6]. The VEGF family of ligands plays its role through cell surface receptor tyrosine kinases, VGFR-1, VGFR-2, and VGFR-3 [7]. VEGFR-2 is the most important one through which VEGF exerts its mitogenic, chemotactic, and vascular permeabilizing effects on endothelial cell [4]. Moreover, VEGF interacts with a family of coreceptors called neuropilins (NRP-1 and NRP-2) [8, 9] that strengthen the link between VEGF and its receptors increasing their biological activity.

Overexpression of VEGF in HNSCC is associated with more advanced disease, increased resistance to cytotoxic agents, and poor prognosis [10–16]. In a meta-analysis of 12 studies including 1002 patients affected by cancer of oral cavity (70.8% of patients), pharynx (15.2%), and larynx (14%), VEGF expression was evaluated, and its positivity was associated with a twofold higher risk of death at 2 years [17].

Hasina et al. [18] demonstrated that there are different molecular mechanisms by which each tumor induce angiogenesis. Using sample collected from patients affected by HNSCC and sample of normal and dysplastic mucosa, they conducted an immunohistochemical analysis and gene expression profiling studies. They studied the expression of cytokines (CK) such as VEGF, IL-8/CXCL8, HGF, and FGF-2 in normal, dysplastic, and pathological tissues. These CK are well-known mediators of HNSCC angiogenesis. The authors observed that normal mucosa generally does not express VEGF, IL-8/CXCL8, FGF-2, and HGF and that, where present, the levels of these CKs are very low compared to dysplastic and pathological mucosa. The same CKs are more frequently expressed and at a higher levels in dysplastic oral mucosa. The incidence and the intensity of expression of VEGF, IL-8/CXCL8, FGF-2, and HGF are highest in HNSCC samples. Moreover, they validated the presence of two different clusters in relation to angiogenesis in HNSCC samples: tumors in Cluster A express high levels of VEGF and FGF-2 and low levels of IL-8/CXCL8 and HGF and are characterized by higher levels of microvessel density than tumors in Cluster B, expressing on the contrary low levels of VEGF and FGF-2 and higher levels of IL-8/CXCL8 and HGF. These data suggest that there are at least two different pathways in inducing angiogenesis in HNSCC. This hypothesis has an important therapeutic implication. In fact we can argue that the inhibition of a specific molecular pathways can block the angiogenesis process, and consequently the tumor growth, only if the target of the therapy is expressed by the tumor cells. In the same study the authors used three different HNSCC cell lines with different levels of expression of VEGF that were inoculated in nude mice. Then they treated the experimental models with anti-VEGF antibody, with nonspecific human IgG antibody, or with PBS (phosphate-buffered saline, a buffer solution isotonic and nontoxic to cells). The growth of tumor with high levels of VEGF was inhibited by anti-VEGF treatment while not influenced by nonspecific IgG or PBS. On the other hand anti-VEGF treatment had limited effects on the growth of tumor with low levels of VEGF. In this case no difference in tumor volume was found compared to those treated with nonspecific IgG or PBS. These data may have very important implications in clinical practice and support the need of better understanding the molecular alterations in each specific tumor in order to better select patients for targeted therapies.

## 3. Effect of Antiangiogenic Agents on Xenograft Models

Several studies report the activity of different molecules directed against the angiogenic process in head and neck models.

For example Miyazawa et al. [19] tested the effect of PTK/ZK (Vatalanib) on the initial stages of head and neck tumor angiogenesis. PTK/ZK is a small molecule inhibitor of VEGF receptors [20, 21]. The molecule has just been tested by Kim et al. [22] in anaplastic thyroid carcinoma xenografts in nude mice, and in that study it inhibited the phosphorylation of VEGFR-2 in the endothelial cells and reduced the microvessel density of the models. Miyazawa et al. tested the effects of PTK/ZK on neovascularization in vitro and in vivo. They inoculated experimental mice with different HNSCC lines and treated them with the oral administration of PTK/ZK or vehicle controls. They showed that animals treated with the small VEGF receptor inhibitor developed low microvessel density compared to those treated with vehicle control. Moreover, the models treated with PTK/ZK had a slower tumor progression than controls, even if the difference was not statistically significant. 

Several preclinical data about the association between anti-EGFR and antiangiogenic treatments [23–25] and between radiotherapy and antiangiogenic drugs [26, 27] have been published recently. Moreover, in the last few years a study conducted by Bonner et al. [28] demonstrated the efficacy of the association of radiotherapy and a target therapy such as an anti-EGFR agent (cetuximab) in locally advanced disease. Few studies investigated the intriguing combination of the three approaches together. This association could be very interesting in clinical practice because the production of VEGF is inhibited, at least in part, by anti-EGFR agents while radiotherapy, through the induction of EGFR production in irradiated cells, can lead to neovascularization. So the combination of these three weapons could have synergistic effects.

In a study published in 2007, Bozec et al. [29] evaluated the efficacy of AZD2171, gefitinib, and radiotherapy. AZD2171 is an inhibitor of VEGFR-2, VEGFR-1, and VEGFR-3 in vitro [30], while gefitinib is an EGFR tyrosine kinase inhibitor with antiangiogenic activity [23]. The effects of the combination of the two drugs on tumor growth and of the combination of the drugs with radiotherapy were tested on human head and neck tumor xenografts. The investigators used a cell line, CAL33, that had high levels of EGFR end VEGF. Mice inoculated with CAL33 tumors were treated with vehicle alone, AZD2171 or gefitinib alone or in combination, or with the two drugs combined with radiotherapy. The treatment with AZD2171 and gefitinib showed better antitumor effects than either treatment alone, but tumor regrowth after discontinuation was observed. On the other hand, the triple combination (two drugs plus radiotherapy) had the best antitumor effects with a prolonged activity after treatment discontinuation. 

The same authors conducted a similar study [31] using bevacizumab, monoclonal antibodies directed against VEGF, erlotinib, an EGFR tyrosine-kinase inhibitor, and radiotherapy on head and neck orthotopic models. They tested the efficacy of the three treatments, given alone or in combination, on mice inoculated with CAL33 tumors. Treatment with each single agent did not show a significant activity on tumor growth while the combination of the three treatments had the best antitumor activity with supra-additive effects (combined ratios 2.3). An evaluation of vascularization marker was conducted in the same study and showed that the triple combination led to a decrease in cell proliferation and neoangiogenesis (lower Ki-67 and VEGFR-2 expression). 

Then the same authors [32] tested the in vivo efficacy of the combination of sunitinib, a multitargeted tyrosine kinase inhibitor with great anti-VEGF activity, cetuximab, and radiotherapy. CAL33 cell lines were injected in mice that were then treated with vehicle or cetuximab and/or sunitinib and/or radiotherapy. In this study the treatments given alone showed a significant antitumor effect compared with controls. The best result on tumor growth was obtained by the triple combination. In fact at the end of the treatment with cetuximab, sunitinib, and radiotherapy, no tumor cells were detectable in all treated animals (*P* < 0.001 versus control).

Myoung et al. [33] conducted a study using the combination of paclitaxel and thalidomide on xenotransplanted oral squamous cell carcinoma. Thalidomide is able to inhibit neovascularization and tumor growth [34–37] while paclitaxel is an antitumor agent with antiangiogenic activity [38–41]. In this study a human oral squamous cell carcinoma line was inoculated into nude mice subsequently treated with thalidomide, paclitaxel, or control vehicle. Paclitaxel showed a significant activity on tumor growth, while thalidomide did not show any effect. It is worthwhile noting that the two drugs had remarkable effects on the immunohistochemical expression of VEGF and CD31, which was also reduced by the administration of paclitaxel and thalidomide. A similar reduction in the production of VEGF mRNA suggested a good activity of these drugs against neovascularization. The study suggests that the inhibition of angiogenesis is not enough to suppress oral squamous cell carcinoma growth and that probably antiangiogenic treatments have to be integrated with other different approaches. 

## 4. Effect of Antiangiogenic Agents in Clinical Trials

Sorafenib and sunitinib are two tyrosine kinase inhibitors with activity against VEGFR2, VEGFR3, and the PDGF receptors that have been tested in different studies in patients with recurrent or metastatic HNSCC.

Three studies were reported with sunitinib. In the first study [42], 22 patients with recurrent or metastatic HNSCC who had received no more than two prior chemotherapy regimens were treated with sunitinib administered in 6-week cycles at 50 mg/day for 4 weeks followed by 2 weeks off. Patients were divided into 2 cohorts according to the Eastern Cooperative Oncology Group Performance Status (ECOG-PS): patients with ECOG-PS 0-1 in cohort A, patients with ECOG-PS 2 in cohort B. the primary endpoint was objective tumor response for group A (15 patients) and feasibility for group B (7 patients). In cohort A partial response (PR) was reported in only one patient, while no response was observed in cohort B. Stable disease (SD) was observed in 25% of patients. The median overall survival (OS) was 21.1 weeks for patients in Cohort A and 19.1 weeks for patients in cohort B. The main grade 3 hematologic toxicities reported were lymphopenia (18%), neutropenia (14%), and thrombocytopenia (5%). The only grade 4 hematologic toxicity observed was thrombocytopenia occurring in one patient. The most common nonhematologic grade 3 toxicities were fatigue and anorexia (23% of patients). Grade 3 hypertension occurred only in one patient in cohort B. Grade 4 hemorrhage was reported in one patient (gastro-intestinal bleeding). Nonfatal hemorrhagic events were seen in 8 patients; in 1 of these patients a superficial tumor bleeding was observed. Even if sunitinib was well tolerated, accrual was closed at interim analysis as nonsignificant antitumor activity was demonstrated.

Another study was conducted by Fountzilas et al. [43] who treated 17 patients affected by metastatic or recurrent HNSCC with sunitinib in first-line setting. The primary endpoint of the study was objective response rate (ORR) while the secondary endpoints included time to tumor progression (TTP), OS, safety, and tolerability of sunitinib as monotherapy. Fourteen patients were assessable for response. Three patients (18%) had stabilization of disease while 11 patients (65%) showed progression. No objective responses were observed. Median TTP was 2.3 months, and median OS was 4 months. The most common grade 3 toxicity was fatigue that occurred in 7 patients (41%), while grade 3 hemorrhagic events were described only in 1 patient (6%). Bleeding of any grade was reported in 10 patients (59%). The study was discontinued because the drug proved to be barely active.

These 2 studies showed that sunitinib 50 mg/day for 4 weeks followed by 2 weeks rest is well tolerated but has no significant antitumor activity in monotherapy.

In the third study [44], sunitinib 37.5 mg daily, given continuously until disease progression or unacceptable toxicity, was tested on 38 patients with recurrent or metastatic HNSCC refractory to platinum-based treatment or unfit for platinum-based regimens. No more than 2 prior lines of chemotherapy were permitted. The primary endpoint was the rate of disease control (RDC), defined as complete response (CR) or PR or SD at 6 to 8 weeks after treatment initiation. RDC was 50% (1 patient with PR and 18 patients with SD). The median PFS was 2 months while the median OS was 3.4 months. The most frequent grade 3/4 toxicities were fatigue (32%), anorexia (16%), thrombocytopenia (13%), and diarrhea (8%). Serious hemorrhagic events of head and neck vessels (grades 3–5) were reported in 5 patients (one grade 3, one grade 4, and 3 grade 5); four of these patients were previously irradiated in the head and neck area. In conclusion this study showed a limited activity of sunitinib in the treatment of recurrent or metastatic HNSCC while reporting a significant risk of severe hemorrhage. 

Sorafenib is the other small tyrosine kinase inhibitor tested in the same setting. It is a multitarget drug with activity against the EGFR-Ras-Raf-Mek-Erk signaling pathway and against VEGF-VEGFR. 

In the first study, published in 2007 [45], Elser et al. conducted a single-arm phase II study in patients affected by recurrent or metastatic HNSCC (including nasopharyngeal carcinoma) that had previously received no more than one systemic treatment. The trial enrolled 28 patients, treated with sorafenib 400 mg twice daily continuously, 27 evaluable for efficacy. The primary objective was ORR. The ORR was 3.7%, while 37% of patients achieved a stabilization of disease as best response. Median TTP was 1.8 months; median OS time was 4.2 months. The most common grade 3 toxicities were lymphopenia (17%) and fatigue (7%).

The other published study, with sorafenib in first-line setting, was conducted on patients with persistent, recurrent, or metastatic HNSCC [46]. The primary endpoint was response probability. Sorafenib was administered as continuous treatment, 400 mg twice daily. Forty-one patients were eligible for response; one patient had a confirmed PR (2%). The estimated median PFS was 4 months, and the estimated OS was 9 months. The most common grade 3 adverse events were hand-foot syndrome (7.3%), stomatitis (4.8%), and nausea (4.8%). The only grade 4 event was a cerebral ischemia caused by asymptomatic pulmonary embolism. 

The two studies demonstrated that sorafenib is well tolerated in this population of patients. No significant activity was demonstrated in terms of response rate, but we must consider that these novel drugs have often a cytostatic effects with limited cytotoxic activity. It is interesting to note that the trials conducted on chemonaive patients showed PFS and OS comparable to those achieved with more toxic and aggressive regimens based on platinum and taxanes. 

A further antiangiogenic agent tested in recurrent and metastatic setting is the monoclonal antibody bevacizumab directed against VEGF. It was administered in association with erlotinib, an anti-EGFR inhibitor, to patients with recurrent or metastatic HNSCC never treated or previously treated with one line of chemotherapy [47]. It was a phase I/II study in which the authors used the association of an anti-vascular agent and an anti-EGFR one; there are in fact several trials in other cancers demonstrating that the use of these type of drugs together improves efficacy [48–53]. The phase I study was designed to determine the maximum tolerated dose of bevacizumab when associated to erlotinib: erlotinib was given at dosage of 150 mg/daily, while bevacizumab was administered in escalating dose cohorts. The primary objective of the phase II study was ORR and TTP; in this phase bevacizumab 15 mg/kg was administered every 3 weeks. Forty-eight patients enrolled were evaluable for response. An objective response (PR or CR) was reported in 7 patients (15%), while 15 patients (31%) maintained stability of disease. Four patients achieved a complete response with a duration of response that lasted up to 17 months in one patient. The median PFS was 4.1 months, and the OS was 7.1 months. The treatment was well tolerated. Grade 3 adverse events reported were esophagitis (1 patient), diarrhea (1 patient), and lymphopenia (1 patient). There was one grade 4 hemorrhage. In this study the association was well tolerated with an interesting activity if compared to trials with antiangiogenic agents used alone. The authors also conducted an exploratory study to investigate biomarkers that could predict clinical outcomes and find that high phosphorylated VEGFR2/VEGFR2 and endothelial cells phosphorylated EGFR/EGFR ratios in baseline tumor specimen can identify patients with the greatest probability of response to erlotinib and bevacizumab.

With regard to locoregionally advanced disease, few studies have just been published. 

Seiwert et al. [54] added bevacizumab to fluorouracil and hydroxyurea-based chemoradiotherapy in patients with relapsed previously irradiated HNSCC or with poor prognosis newly diagnosed disease. It was a phase I study to determine the maximum tolerated dose of bevacizumab when added to chemoradiotherapy. Forty-three patients were enrolled: 29 patients (67.4%) were previously irradiated, while 14 patients (32.6%) were newly diagnosed. Dose-limiting toxicities were reached at level 3 (bevacizumab 5 mg/kg), so at level 4 (bevacizumab 10 mg/kg), the dosage of chemotherapeutic agents alone was reduced. The treatment was well tolerated with grade 3 mucositis occurring in 69.8% of patients and grade 3 radiation dermitis in 11.6%. The adverse events probably related to bevacizumab were grade 3 hypertension in 3 patients, 1 allergic rash reaction, 2 deep vein thrombosis, 1 stroke, and 2 fatal hemorrhages. The median OS of the patients enrolled was 10.7 months. Patients with no prior radiation had a significantly longer OS (40.1 months) than those previously irradiated (10.3 months). This study demonstrates that bevacizumab 10 mg/kg every two weeks can be safely integrated to fluorouracil and hydroxyurea-based concomitant chemoradiotherapy: the rate of severe complication was similar to those reported in trials with different agents in cohorts of patients with the same characteristics [55–68].

Two more interesting studies were presented in the form of abstract at the 2009 ASCO annual meetings as preliminary results.

In the first trial [68] the authors treated 60 patients with newly diagnosed locoregionally advanced HNSCC with two courses of induction chemotherapy repeated every 21 days consisting of carboplatin AUC 6 day 1, paclitaxel 200 mg/mq day 1, 5 fluorouracil 200 mg/mq/day continuous infusion every 3 weeks, and bevacizumab 15 mg/kg day 1 followed by radiotherapy and concomitant paclitaxel 50 mg/mq/weekly, bevacizumab 15 mg/kg weeks 1 and 4, and erlotinib 150 mg daily for 7 weeks. Forty-one patients (85%) completed all treatments with an objective response rate of 77%. This study has a short followup but interesting 18 months PFS of 85% and 18 months OS of 87%. Severe toxicity during induction chemotherapy was neutropenia (46%), neutropenic fever (6%), mucositis (14%), diarrhea (14%), and hand/foot syndrome (11%), while during concomitant treatment severe mucositis was experienced by 76% of patients. 

In another trial [69] bevacizumab 15 mg/kg days 1, 15, and 43 and cisplatin 50 mg/mq days 1, 2, 22, 23, 43, and 44 were added to definitive IMRT in patients with previously untreated, stage III/IV, HNSCC. All patients completed the treatment with a locoregional control rate of 100% (3 patients developed distant metastases). Estimated one-year PFS was 83% and estimated one-year OS 88%. The main severe toxicities were mucositis (76%), nausea (24%), vomiting (17%), neutropenia (41%), hemoglobin (17%), hyponatremia (14%). 

Table 1 summarize the main published clinical trials on anti-angiogenetic drugs.

## 5. Mechanism of Resistance to Antiangiogenic Treatment and Future Direction

In many clinical trials anti-angiogenetic drugs have a limited efficacy, especially in terms of overall survival. Some authors [70, 71] demonstrated in their laboratories that VEGF-targeted drugs inhibit the growth of primary tumors but may shorten survival of mice by promoting tumor invasiveness and the metastatic process. Ebos et al. [70] tested the role of sunitinib in developing metastasis in mice models. They selected sunitinib because of the schedule of administration in clinical practice (4 weeks on/2 weeks off) and the preliminary observations that tumor regrowth can occur during rest period [72]. They showed that sunitinib inoculated in different schedules and doses and with different tumor cell models can lead to opposite results on tumor growth. For example, sustained treatment of pre-established tumors inhibits its growth, while short-term treatment prior to tumor inoculation results in the acceleration of metastasis and reduction in survival. Pàez-Ribes et al. demonstrated in the same issue of Cancer Cells [71] that two different mouse models of tumors, pancreatic neuroendocrine cancer (PNET) and glioblastoma multiforme, can develop an adaptive and evasive response to an efficacious antiangiogenic treatment. This leads to a more aggressive behavior, increased dissemination, and distant metastasis progression. In PNET models two different drugs were tested, sunitinib and a specific VEGFR2 inhibitor. Sunitinib had significantly better efficacy than the competitor but surprisingly led to the development of more invasive tumors.

So, how can these different effects of treatment with angiogenic inhibitors be explained? First of all these agents act by inducing hypoxia, but tumor cells are often able to survive in hypoxic conditions thanks to the ability of producing energy in the absence of oxygen [73]. So hypoxia selects those cells that are more malignant and less sensitive to treatment with these classes of agents [74].

Moreover, tumors can activate more vascular supply mechanisms through upregulation of proangiogenic stromal cells (fibroblasts, pericytes, mesenchimal and hematopoietic cells) that contribute to the vasculature scaffold. Antivascular agents cause acute hypoxia that leads to the accumulation of endothelial progenitors cells at the tumor margins [75]. Both macrophages and neutrophils in proximity of hypoxic tissues can contribute to angiogenesis, escaping the mechanism of action of the drugs [76, 77]. Furthermore hypoxia caused by antiangiogenic treatments causes an increase in bone marrow-derived cells consisting in vascular progenitors and pro-angiogenic monocytic cells (monocytes, hemangiocytes VEGFR-1+ and CD11b+ myeloid cells) [78–82], all involved in the activation of angiogenesis-expressing cytokines, growth factors, and proteases [83, 84]. CD11b+Gr1+ cells are well known for their ability to confer resistance to anti-VEGF in mouse models. These cells derive from bone marrow, are present at high level in tumor and peripheral blood of tumor-bearing animals [85], and produce several angiogenic factors, such as Bv8 [86]. 

In addition VEGF-inhibitors induce an inflammatory state characterized by the production of several cytokines (PlGF, G-CSF, IL-6, erythropoietin, osteopontin) that stimulate angiogenesis and metastasis in a VEGF-independent manner [87]. Another possible mechanism is that anti-VEGF agents or the cytokines induced by their action could inhibit the action of pericytes on tumor vessels, making them more leaky and immature and facilitating the intravasation of tumor cells and metastatic spread [88]. 

The results achieved with antiangiogenic treatment are sometimes controversial, but we must take into account the several variables involved, such as VEGF levels, vessel number, and function, VEGF-dependence of tumor vascularization, pericyte action, recruitment and activation of bone marrow-derived cells, target and duration of treatment, and the combination with different cytotoxic agents. In conclusion it is time to further investigate how to optimally use these agents, with the aim of blocking the tumor growth while suppressing prometastatic effects.

## 6. Discussion

The review of the main studies published in the last years confirms the central role of angiogenesis in the growth and progression of head and neck tumor [18]. Moreover, most of the published data point to the relationship between VEGF overexpression, more advanced disease at diagnosis, and poor prognosis [10–17]. 

Despite the importance of angiogenesis in head and neck cancer, few antiangiogenic agents have shown relevant activity in this clinical setting and have been approved for the treatment of this disease. 

The reason depends mainly on the fact that many studies have been conducted on xenograft models. First of all some data suggest the existence of two different pathways in angiogenesis [18], so it is mostly important to understand as better as possible the pathogenetic process in each patient, in order to select the correct therapeutic target. 

Secondly, the lack of activity can be explained considering that some authors [33] demonstrated that angiogenesis inhibition is probably not enough to completely arrest the growth of tumors; then we should likely combine this approach with cytotoxic drugs or other treatment such as radiotherapy or anti-EGFR agents [29, 31, 32]. 

Furthermore, there are many preclinical data that suggest that antiangiogenic treatments could be effective on primary tumors' growth while promoting the developing of more aggressive disease with a greater prometastatic behavior [70–72].

As for the trials concluded in patients with relapsed or metastatic disease, the drugs that are more extensively studied are TKI inhibitors, Sunitinib and Sorafenib.

Sunitinib [42–44] as monotherapy has shown limited activity in these patients, and so no more studies are warranted. Sorafenib [45, 46] did not give encouraging results with regard to objective response but interesting data of PFS and OS when used as first-line treatment. Few studies have just been concluded and published in patients with locoregionally advanced disease [67, 68], and the results of these trials have to be confirmed with a longer followup.

Finally, it should be interesting to investigate whether the expression of angiogenic factors can be used as predictive. To date only one study [47] with a combination of an antiangiogenic agent and an anti-EGFR inhibitor reported a possible role of a molecular biomarker that could predict a greater possibility of response to an antiangiogenic treatment. Further studies are needed to understand the mechanisms of response and resistance to angiogenesis inhibitors, how to integrate antiangiogenic therapies in the treatments of patients affected by HNSCC, and how to identify those most likely to respond, in order to offer the best treatment for each patient while limiting toxicities.

## Figures and Tables

**Table 1:** Clinical trials with antiangiogenic drugs.

Regimen	Setting	No. of patients	Results
Sunitinib [42]	Recurrent/metastatic (first line)	22	RDC 33% (terminated after interim analysis)
Sunitinib [43]	Recurrent/metastatic (first line)	17	Terminated for lack of efficacy
Sunitinib [44]	Recurrent/metastatic (platinum refractory)	38	RDC 50%. mPFS 2 months, mOS 4 months
Sorafenib [45]	Recurrent/metastatic (second line)	28	ORR 37%. mOS 4.2 months
Sorafenib [46]	Recurrent/metastatic (first line)	41	mPFS 4 months. Estimated OS 9 months
Erlotinib+bevacizumab [47]	Recurrent/metastatic (first/second line)	48	RR 15%. mPFS 4.1 months. mOS 7.1 months
Bevacizumab+fluorouracil+hydroxyurea and radiotherapy [54]	Locally advanced relapsed or poor prognosis newly diagnosed	43	mOS 10.7 months
Carboplatin+paclitaxel+5 fluororuracil+ bevacizumab followed by radiotherapy +paclitaxel+bevacizumab+erlotinib [68]	Locally advanced (first line)	60	ORR 77%. 18 months PFS 85%, 18 months OS 87%
Bevacizumab+cisplatin+IMRT [69]	Locally advanced (first line)	42	Locoregional control rate 100%. Estimated 1 year PFS 83%; estimated 1 year OS 88%

RDC: rate of disease control; mPFS: median progression-free survival; mOS: median overall survival; ORR: overall response rate.
